# Bladder incarceration following anterior external fixation of a traumatic pubic symphysis diastasis treated with immediate open reduction and internal fixation

**DOI:** 10.1186/1754-9493-2-26

**Published:** 2008-10-19

**Authors:** Ryan P Finnan, Michael A Herbenick, Michael J Prayson, Mary C McCarthy

**Affiliations:** 1 Department of Orthopaedic Surgery, Wright State University-Boonshoft School of Medicine and Miami Valley Hospital, Dayton, Ohio, USA; 2 Department of General Surgery, Wright State University-Boonshoft School of Medicine and Miami Valley Hospital, Dayton, Ohio, USA

## Abstract

Anterior pelvic ring disruptions are often associated with injuries to the genitourinary structures with the potential for considerable resultant morbidity. Herniation of the bladder into the symphyseal region after injury with subsequent entrapment upon reduction of the symphyseal diastasis has seldom been reported in the literature. We report such a case involving bladder herniation and subsequent entrapment after attempted closed reduction with anterior pelvic external fixation immediately treated with open reduction and internal fixation along with a review of the literature.

## Background

Pelvic fractures are a small but clinically significant percentage of all fractures. Associated injuries to the genitourinary structures ranging from urethral and prostatic injuries to complete bladder rupture with resultant morbidity have been described in the literature [1-5]. The use of anterior pelvic external fixation has been shown to be a reliable and effective means to stabilize pelvic injuries in the acute resuscitative phase of the trauma patient [6-9]. However, urologic injuries, particularly bladder entrapment, remain a concern with the use of closed reduction of symphyseal disruption and anterior pelvic external fixation [10,11]. We report a case involving bladder herniation through a traumatic symphyseal diastasis with subsequent incarceration after attempted reduction with pelvic external fixation and a review of the literature.

## Case presentation

**Figure 1:**
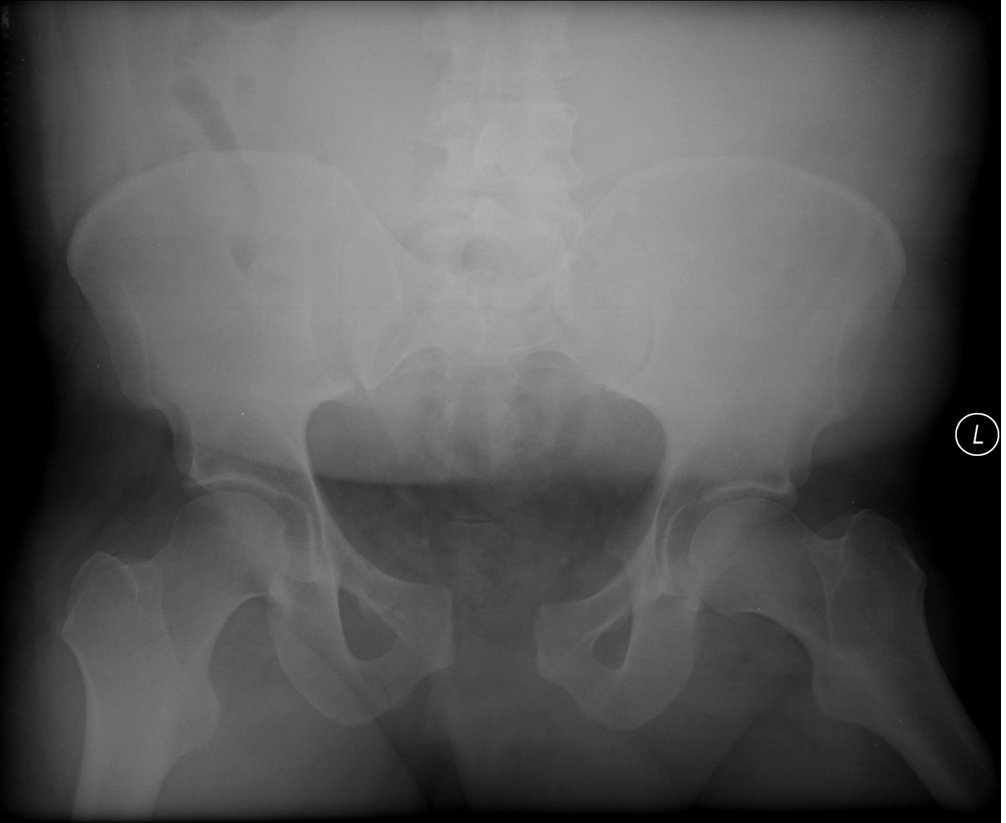
Presenting AP pelvis radiograph demonstrating the open-book pelvic fracture with right, nondisplaced superior and inferior rami fractures, symphyseal diastasis of 4 cm and complete disruption of the right sacroiliac joint.

**Figure 2:**
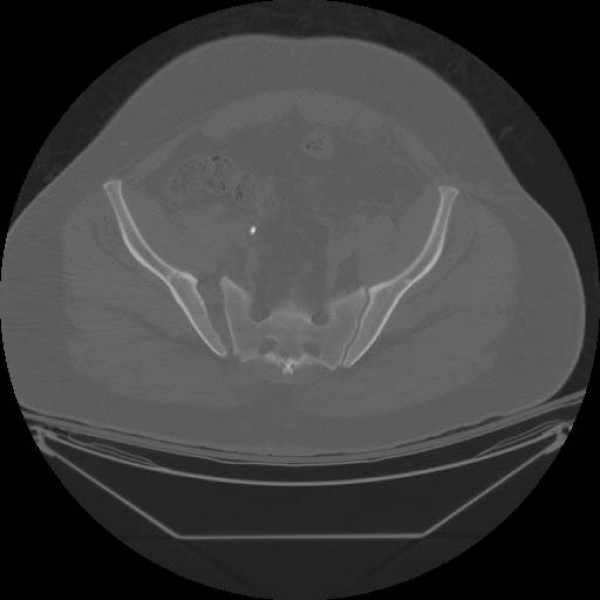
Presenting representative axial pelvic CT cut showing complete right sacroiliac joint disruption.

A thirty-eight year old male presented to our institution after involvement in a head-on motor vehicle collision as a restrained driver. He was hemodynamically stable on admission, and multiple injuries were identified including an unstable open-book pelvic ring disruption (right, non-displaced superior and inferior ramus fractures, a symphyseal diastasis of 4 cm and complete disruption of the right sacroiliac joint; Young-Burgess [12] APC III; Tile [13] C1.2) (Figure 1: Presenting AP pelvis radiograph demonstrating the open-book pelvic fracture with right, nondisplaced superior and inferior rami fractures, symphyseal diastasis of 4 cm and complete disruption of the right sacroiliac joint. and Figure 2: Presenting representative axial pelvic CT cut showing complete right sacroiliac joint disruption.). Additional injuries included a left type IIIA [14] open femur fracture, a right patella fracture, a right fifth metacarpal fracture, a right talar lateral process fracture and a right calcaneal fracture.

**Figure 3:**
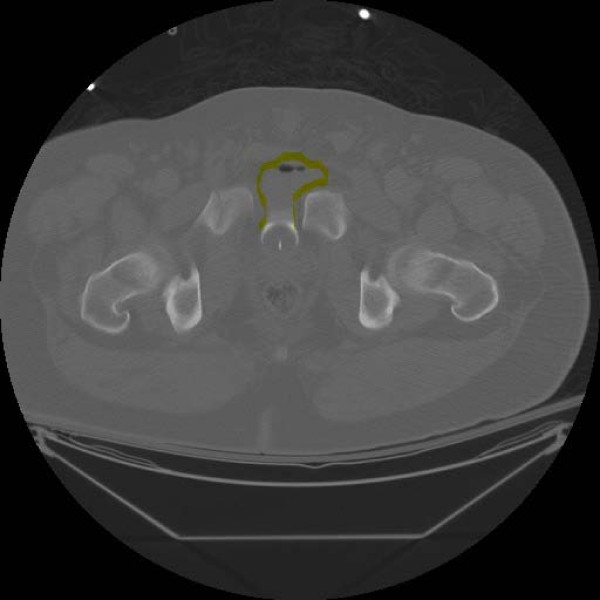
**Presenting representative axial pelvic CT cut showing herniation of the urinary bladder into the diastatic symphysis.** The herniated bladder has been outlined for clarity.

Primary survey of the patient revealed no blood at the urethral meatus and a normal prostate. A Foley catheter was inserted without difficulty and returned clear urine. The catheter was clamped to distend the bladder for enhanced visualization on subsequent computed tomography (CT) scan of the abdomen and pelvis. Admission urinalysis showed 100–150 red blood cells/high-power field. The presenting CT scan of the abdomen and pelvis showed bladder herniation into the symphyseal diastasis (Figure 3: Presenting representative axial pelvic CT cut showing herniation of the urinary bladder into the diastatic symphysis. The herniated bladder has been outlined for clarity.). Upon completion of the CT scan, the clamped catheter was released to decompress the bladder.

**Figure 4:**
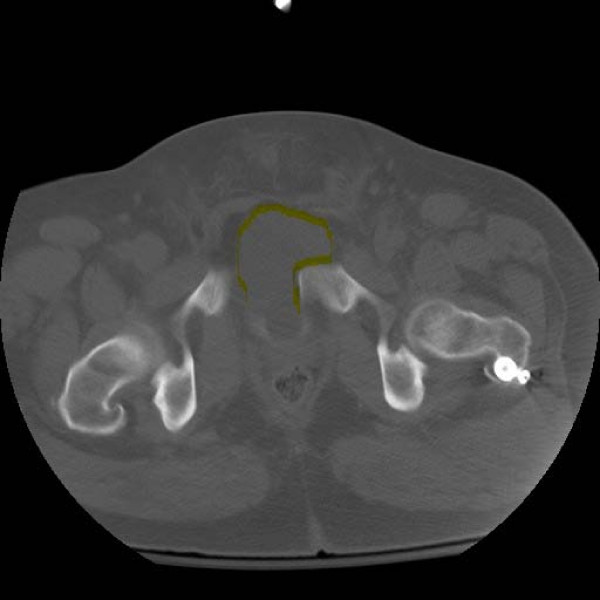
**Postoperative CT cystogram showing incomplete reduction of the symphysis with persistence of the bladder incarceration within the pubic symphysis.** The herniated bladder has been outlined for clarity.

After clearance from the general surgery trauma team, the patient was taken to the operating room for irrigation and debridement of the open fracture wound and antegrade intramedullary nailing of the left femur. Provisional pelvic stabilization was achieved with the application of an anterior pelvic external fixator. An attempt at manual reduction was incomplete but accepted with mild residual rotational displacement of the right hemipelvis. Some resistance to full reduction was noted by the treating orthopaedist. Referral to an orthopaedic traumatologist was made and a postoperative CT scan was obtained. This revealed incomplete reduction and persistence of the bladder incarceration within the pubic symphysis (Figure 4: Postoperative CT cystogram showing incomplete reduction of the symphysis with persistence of the bladder incarceration within the pubic symphysis. The herniated bladder has been outlined for clarity).

**Figure 5:**
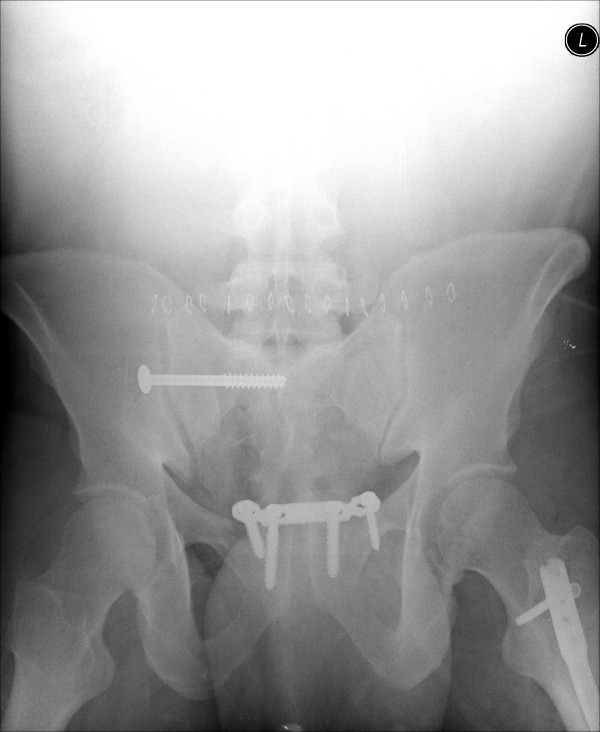
Immediate postoperative outlet radiograph showing satisfactory symphyseal and sacroiliac joint reduction and fixation.

The patient was returned to the operating room later the same day by the orthopaedic traumatologist for removal of the external fixator, open reduction and internal fixation of the pubic symphysis dislocation, and percutaneous screw fixation of the right sacroiliac joint. During the anterior pelvic fixation through a Pfannenstiel incision, the bladder was noted to be intact and was easily reduced. During the course of the pelvic ring reduction, the ramus fractures were visualized with fluoroscopy and remained non-displaced. Thus, a decision was made not to extend the internal fixation beyond the ramus fractures. The symphysis was reduced and fixed under direct visualization using a four-hole symphyseal plate (Zimmer, Warsaw IN) and large fragment screws. The right sacroiliac joint was reduced and stabilized percutaneously with a 7.3 mm cannulated screw and washer (Synthes, Paoli PA). A postoperative radiograph revealed a near-anatomic reduction of the symphyseal and sacroiliac dislocations (Figure 5: Immediate postoperative outlet radiograph showing satisfactory symphyseal and sacroiliac joint reduction and fixation).

**Figure 6:**
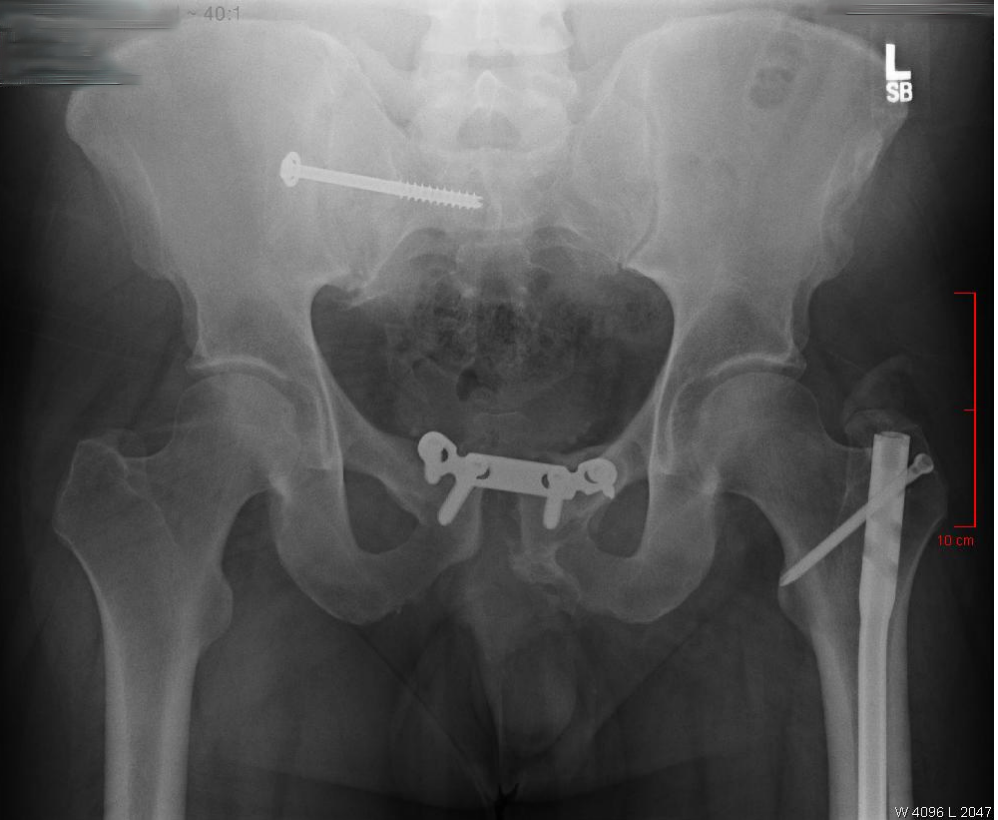
12 month follow-up AP pelvis radiograph showing mild loss of symphyseal reduction.

At one year post-injury, the patient was doing well. He had no urinary complaints, but reported moderate discomfort with sexual activity. Follow-up radiographs revealed further residual displacement of the symphysis and SI joint without obvious failure to the plate or screw fixation (Figure 6: 12 month follow-up AP pelvis radiograph showing mild loss of symphyseal reduction.). No intervention was felt necessary secondary to minimal clinical symptoms from the pelvis. His 12-month Short Musculoskeletal Function Assessment produced a Dysfunction Index of 27.94 and a Bother Index of 31.25 (versus normative population means of 12.70 ± 15.59 and 13.77 ± 18.59 respectively) [15,16]. His 12-month SF-36 Physical and Mental Component Summaries were 34.3 and 36 respectively (versus normative population means of 50 ± 10) [15,16].

## Discussion

A review of the literature returned nine previous reports of bladder herniation through a traumatic symphyseal diastasis, only two of which involved actual bladder incarceration after anterior external fixation [10,11,17-23]. The first report by Fuhs and associates [17] describes a patient treated in a pelvic sling with initial adequate reduction of the symphyseal diastasis. Persistent, intermittent microscopic hematuria and eventual gross hematuria one year after the injury led to open reduction and internal fixation with intraoperative findings of pubic bone erosion through the bladder wall.

Cass and associates [18] reported two cases involving bladder problems with pelvic external fixation. One case involved the acute reduction of both the bladder herniation and symphyseal disruption with external fixation. Six months post-injury, the diastasis recurred and bladder herniation was found at the time of open reduction and internal fixation eight months after the injury. The authors recommended intra-operative inlet-view cystograms with external fixator symphyseal reduction and consideration of internal fixation. Neser and Lindeque [19] also warned against the possibility of interposed bladder and soft tissue with open-book pelvic injuries. They reported symphyseal diastasis that was irreducible with multiple closed attempts and found interposed bladder and perivesicular soft tissue at the time of open reduction and fixation.

Cespedes and colleagues [20] reported spontaneous reduction of a bladder herniation through a 3.5 cm pubic diastasis. Microhematuria was present on admission and a cystogram showed the herniated bladder. The patient refused to undergo the planned open reduction, and one week later a voiding cystourethrogram revealed spontaneous reduction of the bladder herniation. The patient remained asymptomatic at four months.

Only two of these five reports actually describe incarceration of the bladder after anterior external fixation and reduction of a pubic diastasis. Bartlett and colleagues [10] reported the case of a man initially treated with anterior pelvic external fixation for an open-book pelvic injury. The entrapped bladder was recognized with a postoperative CT cystogram and re-manipulation of the pelvis and fixator failed to reduce the incarcerated bladder. The patient underwent open reduction and fixation of the pubic symphyseal diastasis 10 days post-injury. Persistent bladder incarceration was noted and reduced. Gerraci and Morey reported a similar case where closed reduction and external fixation of the pelvic fracture were performed in an unstable multi-injured patient11. Twenty-four hours later, postoperative CT revealed bladder entrapment in the reduced pubic diastasis. Definitive internal fixation was performed without complication.

The use of anterior pelvic external fixation has been shown to be a reliable and effective means to stabilize pelvic injuries in the acute resuscitative phase of the trauma patient [6-9]. Our patient presented with 4 cm of pubic symphysis diastasis and disruption of the right sacroiliac joint. Injury to the supporting soft tissue structures (puboprostatic and pubovesical ligaments and pelvic fascia) is expected for bladder herniation to occur [4]. With adequate reduction and stabilization, these structures heal by scar tissue [24]. However, interposition of soft tissue within the pubic symphysis impedes healing and potentially leads to late widening, as in the cases reported by Fuhs et al [17] and Cass et al [18].

Signs of urological injury include blood at the urethral meatus, a high-riding prostate gland, and gross and microscopic hematuria [3,4]. If signs of urological injury are present, retrograde urethrography prior to Foley catheter insertion is commonly performed. However, the detection of lower urologic injuries can be difficult. Ziran et al [5] reported that 23% of bladder and urethral disruptions associated with pelvic fracture were initially missed in their series of 43 patients. In this case, only microhematuria was noted on presentation, and the urinary catheter was placed without difficulty. Bladder herniation was first noted when the patient underwent abdominal and pelvic CT scanning. However, because the scan was obtained with the catheter clamped and the bladder distended, it was felt that decompression of the bladder upon release of the clamp would allow reduction of the herniation.

In our case, adequate reduction of the symphyseal disruption was not obtained by closed means. Immediate postoperative CT scanning of the pelvis showed persistent herniation, which was addressed through formal open treatment. We agree with previous recommendations that when difficulty in obtaining a closed reduction is experienced, incarcerated soft tissue should be considered [10,11,20].

## Conclusion

Although bladder herniation into a traumatic pubic symphyseal disruption is rare, an index of suspicion is warranted. If herniation is observed preoperatively and the patient's status allows, consider direct open reduction and internal fixation. If an external fixator or pelvic binder is used, then a post-operative CT cystogram should be obtained shortly thereafter to confirm bladder position. If incarceration is identified, timely open reduction and anterior ring stabilization are recommended to minimize the risk of bladder necrosis or perforation.

## Competing interests

The authors declare that they have no competing interests.

## Authors' contributions

All authors, RPF, MAH, MJP and MCM, were involved in the clinical care of the described patient. RPF wrote the initial manuscript and all revisions. MAH, MJP and MCM all contributed in revising the manuscript critically for scientific and clinical content and gave final approval.
